# *Notes from the Field:* Outbreak of Ebola Disease Caused by Bundibugyo Virus — Democratic Republic of the Congo and Uganda, May 2026

**DOI:** 10.15585/mmwr.mm7522e3

**Published:** 2026-06-11

**Authors:** Delayo L. Zomahoun, Mary Adetinuke Boyd, Margaret A. Honein, Joel M. Montgomery, Emily Zielinski-Gutierrez, Elissa Meites, Athalia Christie, Satish K. Pillai, CDC 2026 Ebola Response

**Affiliations:** ^1^CDC 2026 Ebola Response, Atlanta, Georgia; ^2^CDC country office, Kinshasa, Democratic Republic of the Congo; ^3^CDC country office, Kampala, Uganda; ^4^U.S. Public Health Service, Rockville, Maryland.

**Summary**

**What is already known about this topic?**

Bundibugyo virus has caused two previous Ebola disease outbreaks in the Democratic Republic of the Congo (DRC) and Uganda.

**What is added by this report?**

In May 2026, a large outbreak of Bundibugyo virus disease was identified in DRC and Uganda. As of June 2, a total of 378 confirmed cases and 63 confirmed deaths have been reported. No cases have been reported in the United States.

**What are the implications for public health practice?**

To help reduce the risk for continued spread of Bundibugyo virus, including potential spread beyond DRC and Uganda or importation to the United States, ongoing collaboration between CDC and international partners and coordination among U.S. government agencies are essential.

Bundibugyo virus disease (BVD) is a type of Ebola disease, a severe and often fatal viral hemorrhagic fever (*1*). Bundibugyo virus was first identified in 2007, when it caused an outbreak in Uganda with 149 suspected cases and 37 deaths (*2*). A 2012 BVD outbreak in DRC resulted in 56 laboratory-confirmed cases and 17 deaths (*3*). On May 15, 2026, the ministries of health in the Democratic Republic of the Congo (DRC) and Uganda declared outbreaks of BVD. As of June 2, a total of 378 confirmed cases and 63 confirmed deaths have been reported.

## Investigation and Outcomes

### Characteristics of Patients

The initial clusters of BVD cases were identified among health care workers in DRC, whose signs and symptoms included acute fever, vomiting, diarrhea, and, in some cases, bleeding (*4*). As of June 2, a total of 378 confirmed cases (363 in DRC and 15 in Uganda) and 63 confirmed deaths (62 in DRC and one in Uganda) have been reported, primarily among adults aged 18–49 years, with cases approximately evenly distributed between females and males. Uganda’s outbreak has primarily involved travelers arriving from DRC, with secondary transmission to health care workers.

### Laboratory Findings

Laboratory analysis by the DRC National Institute of Biomedical Research confirmed Bundibugyo virus (species *Orthoebolavirus bundibugyoense*). Initial genomic sequencing was consistent with a new spillover event (i.e., transmission of virus from its natural reservoir to an intermediate animal) from an unknown zoonotic host.

### Transmission and Treatment

Based on evidence from other Ebola disease outbreaks, Bundibugyo virus is likely transmitted through direct contact with body fluids of an infected person (e.g., blood, vomitus, feces, urine, tears, sweat, saliva, breast milk, amniotic fluid, vaginal secretions, or semen). The incubation period is expected to range from 2 to 21 days, and patients are considered most infectious in the late stages of the disease and after death, when high concentrations of virus are present in body fluids (*1*). Treatment consists of supportive care; no medications or vaccines against BVD have been approved.

### BVD Exposures Among U.S. Citizens

After developing symptoms, one U.S. health care worker in DRC received a positive test result for Bundibugyo virus and was transported to Germany for treatment. Six other U.S. citizens (health care workers and their close contacts) who had high-risk exposures to BVD in DRC were transported to Germany and Czechia for monitoring. No BVD cases have been reported in the United States. This activity was reviewed by CDC, deemed not research, and conducted consistent with applicable federal law and CDC policy.*

## Preliminary Conclusions and Actions

On May 17, 2026, CDC initiated a public health emergency response to support U.S. preparedness, the international outbreak response, and U.S. public health response coordination. The same day, the World Health Organization determined this outbreak to be a public health emergency of international concern. On May 18, the U.S. Department of Homeland Security and CDC announced new public health measures that included temporary U.S. entry restrictions. On May 19, CDC released a health advisory, initiated enhanced airport screening, and issued interim guidance for U.S. health departments managing travelers in their jurisdictions. CDC also issued a Level 3 Travel Health Notice for DRC (reconsider nonessential travel to provinces with cases) and a Level 2 Travel Health Notice for Uganda (practice enhanced precautions).

To reduce the risk for Bundibugyo virus transmission in the United States, CDC is providing outreach and preparedness information for the public, clinical guidance for health care providers, and guidance for state, tribal, local, and territorial partners on public health management. In addition, CDC’s Laboratory Response Network is supporting diagnostic testing capacity at more than 40 U.S. laboratories. When needed, CDC offers clinical consultation for suspected Ebola cases and exposure risk assessments for U.S. citizens abroad who are returning to the United States.

To reduce the risk for spread to other countries and regions, CDC is collaborating with international partners and country offices in DRC and Uganda by providing assistance with epidemiologic investigations and contact tracing, laboratory testing, data management, infection prevention and control, border health surveillance, and risk communication and community engagement. In addition, CDC has worked with international partners to complete readiness assessments in bordering countries.

To support coordination among U.S. government agencies, CDC launched an Ebola dashboard within the Interagency Readiness and Response Hub, a secure collaboration platform, and is providing technical recommendations for BVD diagnostics to the U.S. Department of State. CDC is also collaborating with the Administration for Strategic Preparedness and Response and the National Institutes of Health to guide interagency recommendations on medical countermeasures for BVD.

This ongoing BVD outbreak is occurring in geographic areas that have limited public health infrastructure and are affected by armed conflict, frequent population displacement, and cross-border movement (*5*). The scope of the outbreak is likely larger than that represented by available data and might prove challenging to contain and control.

## Footnotes

* 45 C.F.R. part 46, 21 C.F.R. part 56; 42 U.S.C. Sect. 241(d); 5 U.S.C. Sect. 552a; 44 U.S.C. Sect. 3501 et seq.
